# The Improved Cæsarean Section

**Published:** 1890-02

**Authors:** W. D. Haggard

**Affiliations:** Nashville, Tenn.


VOL. VI. FEBRUARY, 1890. No. 12.

### Original Communications.

### THE IMPROVED CÆSAREAN SECTION.*

BY W. D. HAGGARD, M. D., NASHVILLE, TENN.

To the obstetric surgeon, but more particularly to the parturient woman and her unborn child, the subject is one of commanding importance and will be now calmly and dispassionately considered.

This paper is not intended to be a compilation from medical records or statistical evidences, but is rather iutended to quicken the active and growing sentiment against the various operations which involve the destruction of the living child, a practice, though repugnant to all the better instincts of our nature, that has received the sanction of such high authorities that it was almost the universal practice for centuries.

Taking a retrospective view of the whole field of obstetric medicine, with the facts now beefore us, we think much can be done for the good of humanity.

*Read before the Southern Surgical and Gynecological Association held in Nashville, November 12, 1889.

In action it has reached its fullest capacity for work. In achievement it has never reached the fulness of its power.

The great difficulty which has confronted us as practitioners has been the want of adequate knowledge as to the possibilities which the recent improvements, with increased knowledge, better facilities, better instruments, more perfect technique, and a greater confidence in the outcome of all capital operations, have inspired.

It is a conceded fact that the greatest obstacle to delivery, in an overwhelming majority of cases, is some deformity of the pelvis.

When the obstruction is insurmountable, its nature and extent should be determined before any attempt at artificial delivery is attempted. It is evident that our surgical endeavor will be most intelligently and and effectually applied when based upon a full and accurate knowledge of the nature and the extent of the obstacle to delivery.

That we may obtain a clear and comprehensive conception of the best means at our command to overcome the difficulty, it is necessary to consider the claims which the forceps, version, craniotomy, gastro-clytrotomy, the improved Cæsarean section, the utero-ovarian amputation or the total extirpation of the uterus has to priority, with due regard to the life of both *mother* and *child*.

The sentence of condemnation which was passed upon woman, relating to the tree of knowledge, was that "in sorrow she should bring forth children." This literally means that, notwithstanding all the enchantment of beauty, the joys of love and the delights of the nupital bed, the portal of life is presided over by anguish and danger.

Around the beauteous love of maternity the pangs of anguish ever hang. The sharp cry of the parturient mother, borne upon every breath of air, rings out around the world as it has from the beginning and will until the end.

To prevent this anguish we have yet no remedy. The angel of anæsthesia, indeed, hangs over the couch of motherhood, but the pangs and perils still abide, exciting the sympathy of the accoucheur in behalf of the mother, while the child comes into the world sprinkled with its mother's blood, receiving its life at the jeopardy of hers, even in normal uncomplicated labor. But what horror and responsibility crowds around the couch of motherhood, when nature has not her usual course by reason of mal-presentation, pelvic distortion, or other obstruction to labor, which come in as factors of suffering, danger and death!

Here the accoucheur has a responsibility second to none in the whole range of human duty. Two lives hang upon the grappling hook of his judgment. To stand worthily by the couch of the parturient woman with these invironments, he must be thoroughly equipped with diagnostic skill, accurate anatomical knowledge, and mechanical ability, preceded by profound study, yet he must have a sense of conscientious duty and consider well between the destructive operations of the past, and the conservative methods of to-day.

Between these two operations much difference of opinion has ever existed. Therefore, he must decide between them, and at the peril of human torture, danger and, it may be, death.

When Baron Larrey approached the great Napoleon during the labor of Marie Louise, with the question as to which life the Emperor desired to be saved, in the event the dread decision was forced upon them, Napoleon replied: "Do not be alarmed, regain your self-possession and professional nonchalance. Forget that you are accouching the Empress and giving birth to the King of Rome. Treat my wife as you would a Bourgeoise of the Faubourgs. Then, if one life must be sacrificed, it is the mother's right to life that must prevail; she must have the preference."

The result is historical; doubtless Napoleon's coolness and nerve restored courage to the physician and contributed to the result.

The wonderful influence which the great name of Napoleon wielded in his day doubtless contributed to the general and sweeping canon that the mother has the prior and superior right to life, but this opinion does not commend itself to the unqualified acceptance of the medical profession, nor by our brothers of the sacerdotal cloth. The great Catholic church of imperial

Rome, that great conservator of force, that most ancient of all forms of polity, coming down to us through so many centuries, and so full of human impulses, and yet regardful of the sacred soul that is in both fœtus and mother alike, affirms through the Theological Faculty of Paris, "that if it is not possible to extract the infant without killing it, it is not possible to extract it without mortal sin." I am in full sympathy with the humane impulse which prompted this statement, and deplore the obstetric dictum which required that the fœtus should be sacrificed to save the mother. Life is the gift of God, and no man has the right to destroy one life to save another except it be in self-defence. So long as the life of the mother was made to depend on the death of the fœtus, but little advance was made in prolonging the one, or preserving the other.

The advance of the art and science of obstetrics no longer justifies the needless sacrifice of hecatombs of unborn children. The first right of the child is to be born. It may seem utopian to demand that the right be respected, but as Cæsar paused on the brink of the Rubicon, so should others pause ere they say the "die is cast." In remote antiquity many women perished in the travail of labor without artificial aid; but as the science and art of obstetrics advanced, surgeon-midwives attempted various expenditures.

Tradition has it that Julius Cæsar received his surname by being, like the charm destroying MacDuff, "from his mother's womb untimely ripped." During the middle ages the operation of craniotomy so greatly increased that version was a "lost art," and the forceps were, for the time, completely buried in oblivion.

About the time Bandelocque and his contemporaries flourished, craniotomy was recognized as a highly scientific procedure, and the destructive operations were practiced with such apparent recklessness that when its results were considered, they were too appalling to contemplate. Forty to fifty per cent. rates of mortality are allowed by him and Rokitansky to have followed. Playfair says, after alluding to the great destruction of fœtal life, that: "We could not look back to it without a shudder," and asserting that "craniotomy was performed three or four times as often as forceps delivery;" that, "fortunately, professional opinion has completely recognized the sacred duty of saving the infant's life whenever it is practicable to do so; and British obstetricians now teach, as carefully as those of other nations, the imperative necessity of using every endeavor to avoid the destruction of fœtal life." Again, he says that, "at one period out of 21,867 births in the Dublin Rotunda Hospital, the forceps were never once applied."

Still later, it is certain craniotomy was performed three or four times as often as the forceps were imployed. Yet, notwithstanding such positive statements, child murder has been, too often, the order of the day, since their enunciation; and until the advent of antiseptic methods, the death rate for mothers after embryotomy was fearful; since which time, however, it has been reduced to almost *nil.*

With the revival of Cæsarean section by Sanger, his improvements in the technique of the operation, and the use of antiseptic methods, the light of day began to dawn upon the bloody path of craniotomy, and it has continued to grow brighter until it seems likely to culminate in the acceptance of the improved Cæsarean section, where the child is living and the obstruction to delivery is insurmountable, thus lifting from the shoulders of the obstetricians the terrible yoke they have so long borne.

To deliberately sacrifice the life of the child with the hope of saving that of the mother should long since have been relegated to the realms of desperate expedients, wholly unjustifiable.

The pathetic emphasis of the illustrious Meigs, who refused to perform embryotomy for a historic patient for the third time, sinks deep into the heart of every true philanthropist.

The success of Gibson in delivering her of living children by the Cæsarean section, before it had reached its present perfection, comes to us as a psalm of life and a song of victory, foreshadowing that the hosts of science would, at last, cross the Red sea, in whose waves of blood they had waded so long.

When the improved Cæsarean section was first brought to the notice of the profession by Sanger, in 1880, it seemed as if he had captured, by a sudden dash, the grim outworks of the citadel of death, so long frowning upon the birth of life.

All over Europe and America antiseptics, the abbreviated name of the angel of cleanliness is hovering over the bed of maternity, protecting both mother and child from many of the ills that flesh is heir to. In the Cæsarean section the office of the physician is to save the life of mother and child. He is not the executioner. That life which he did not give, and which he must not take, is left in the keeping of its author, the Almighty God, in whose hands, in spite of the utmost medical skill, are the tissues of life and death.

The killing of children, at the call of scientific necessity, was always of doubtful propriety and a temptation to reckless and destructive methods that has always borne bad fruit. Who has the right to decide which of two living beings shall die? Who ever exercised such a prerogative with perfect assurance that he was right?

The improved Cæsarean section offers justifiable means of saving both mother and child, and relieves the heart and conscience from the charge of scientific murder.

Embryotomy, on a living child, will soon cease to be regarded as scientific or even a justifiable operation. This seems to be foreshadowed by the statistics of Caruso, who reports a case in detail by Sanger and one by Zweiffel, and adds statistics up to October, 1888, comprising 135 cases. Six successful cases were known in addition to Caruso's, but the details necessary for publication were lacking.

German operators have performed seventy-four of these operations; Americans, eighteen; Austrians, sixteen. The results obtained by Americans are inferior to those of the Germans and Austrians. The results show 74.44 per cent. of recoveries among mothers in all cases, and 91.72 per cent. of recoveries among children. In three cases in which the operation was done the second time, both mothers and children recovered.

It may, therefore, be said that a mother has three chances out of four and her child nine out of ten for life with this operation. A careful estimate of the results of craniotomy under antiseptic precautions shows that 93.04 per cent. of mothers recover. Selecting similar cases on which section was performed, the percentage of recoveries in these cases was 89.04, and 100 per cent. of children.

Caruso concludes, therefore, that craniotomy on the living child is to be superseded by the conservative operation.

From the foregoing estimate I deduce the following:

Given one hundred cases of obstructive delivery, requiring either the destructive or the conservative operation; by the former ninety-three lives will be saved; by the latter one hundred and sixty-five, only a difference of seventy-two lives saved by the Cæsarean section of one hundred deliveries. Statistics, favorable as they are, I believe will, in the near future, show still greater success when the operation by the improved Cæsarean section becomes more fully established. Because, in many cases it is now resorted to as a necessity and not as a choice, after the mother has been exhausted by ineffectual efforts at delivery, when obstruction rendered it impossible. When the obstetrician discovers the obstruction to be insurmountable, he will not hesitate or delay, but select one of the conservative methods and perform it with every advantage that such an operation would have over one forced upon him after the parturient mother has been stretched upon the rack until strength and hope have almost fled.

By the revival of the Cæsarean section an intelligent foundation has been laid as a basis upon which the true philanthropist can labor in behalf of children yet unborn. False views have bridled conscience for centuries. Let science dictate for a while and when results are known men and angels will rejoice.

Wise conclusions are always based on accurate knowledge, the results of which can only be reached by scientific methods. Before conclusions can be effectual the knowledge must be public; it cannot be technical with the public, but it must be general, and relate to the results which have been reached.

The public and the profession have always accomplished much when working together. When this is effected still greater achievements may be won, and the destruction of an unborn child will be known no more in the land. The outcome of the general adoption of the improved Cæsarean section, with its substitutes and various improvements for the destructive operations on the living child, would add immeasurably to the span of human existence, and render the cry of distress and wail of bereavement still less, until in swelling chorus will be heard the merry laugh of children, the sweet voice of young men and women, and the triumph of older men and matrons, saved to the world by the interposition of science.

The requirements of the accoucheur in this "the scientific period" are high and difficult of attainment, but they are such as to excite the ambition of youth, and the emulation of genius. Once attained, he confidently enters the lying-in chamber with wisdom to differentiate and nerve to act in the interest of both mother and child. While his patient is still full of hope and courage he will relieve her of her burden with the keen blade of scientific mercy, and snatch her child from the domain of death, thus giving maternity and childhood alike the same chance for life, condemning neither; thus taking from the sacrificial surgeon the blood of babes.

No Herods will be known reeking with the massacre of the innocents in the halcyon days to come.

